# Tobacco industry issues management organizations: Creating a global corporate network to undermine public health

**DOI:** 10.1186/1744-8603-4-2

**Published:** 2008-01-17

**Authors:** Patricia A McDaniel, Gina Intinarelli, Ruth E Malone

**Affiliations:** 1 Department of Social and Behavioral Sciences, School of Nursing, University of California, San Francisco, CA 94143-0612, USA

## Abstract

**Background:**

The global tobacco epidemic claims 5 million lives each year, facilitated by the ability of transnational tobacco companies to delay or thwart meaningful tobacco control worldwide. A series of cross-company tobacco industry "issues management organizations" has played an important role in coordinating and implementing common strategies to defeat tobacco control efforts at international, national, and regional levels. This study examines the development and enumerates the activities of these organizations and explores the implications of continuing industry cooperation for global public health.

**Methods:**

Using a snowball sampling strategy, we collected documentary data from tobacco industry documents archives and assembled them into a chronologically organized case study.

**Results:**

The International Committee on Smoking Issues (ICOSI) was formed in 1977 by seven tobacco company chief executives to create common anti-tobacco control strategies and build a global network of regional and national manufacturing associations. The organization's name subsequently changed to INFOTAB. The multinational companies built the organization rapidly: by 1984, it had 69 members operating in 57 countries. INFOTAB material, including position papers and "action kits" helped members challenge local tobacco control measures and maintain tobacco-friendly environments. In 1992 INFOTAB was replaced by two smaller organizations. The Tobacco Documentation Centre, which continues to operate, distributes smoking-related information and industry argumentation to members, some produced by cross-company committees. Agro-Tobacco Services, and now Hallmark Marketing Services, assists the INFOTAB-backed and industry supported International Tobacco Growers Association in advancing claims regarding the economic importance of tobacco in developing nations.

**Conclusion:**

The massive scale and scope of this industry effort illustrate how corporate interests, when threatened by the globalization of public health, sidestep competitive concerns to coordinate their activities. The global network of national and regional manufacturing associations created and nurtured by INFOTAB remains active, particularly in relation to the recently negotiated global health treaty, the Framework Convention on Tobacco Control. Policymakers should be aware that although these associations claim to represent only national or regional interests, they are allied to and coordinated with a confederation of transnational tobacco companies seeking to protect profits by undermining public health.

## Background

Globalization, the "increased interconnectedness of peoples and nations through technology, trade, and finance," has the potential to improve or impede public health [1,2]. The globalization of commercial cigarette promotion and the ensuing global epidemic of tobacco-related disease illustrate negative aspects of globalization, and show how globalization's costs may be distributed unevenly between developed and developing nations [3]. One-third of the global population age 15 and over smokes, with the vast majority (84%) living in developing and transitional economy countries [4]. Tobacco is the second major cause of death in the world, killing 5 million people in 2006 [5]. If current smoking trends continue, it is estimated that by 2020 tobacco will kill 10 million people every year, with 70 percent of the deaths occurring in developing nations ([6], p. 38).

Transnational tobacco companies have played a major role in this unfolding public health disaster. During the last half of the twentieth century, knowledge of the risks of tobacco use led to increased regulation and declining consumption in western nations ([7], p. 452). In response, tobacco companies expanded their international operations and supported trade liberalization policies, bringing sophisticated and aggressive marketing techniques to countries with few smoking restrictions and limited knowledge of the health consequences of smoking ([7], pp. 452–3, [8], p. 15, [9,10]). They also developed common strategies to thwart tobacco control efforts at national and regional levels and to maintain tobacco-friendly environments, particularly in developing countries. These strategies were developed by a series of cross-company "issues management" organizations, and implemented through a network of national manufacturers' associations that the transnationals established around the globe.

Although previous research has highlighted some of their activities [11-15], the organizations remain poorly understood, and no previous work has attempted to comprehensively enumerate their projects. This study uses internal tobacco industry documents to describe more fully these issues management organizations and their efforts to undermine public health and advance tobacco industry interests globally. More widespread understanding of their origins, structure, aims, activities, and continuing influence may help protect current and future tobacco control efforts, including the recently negotiated international public health treaty, the Framework Convention on Tobacco Control (FCTC), from tobacco industry interference.

This study adds to the growing literature that draws upon previously secret tobacco industry documents to understand the inner workings of the industry [16,17]. Previous research has, among other things, revealed how the industry has deceived the public and policymakers about the harms of tobacco [18,19], manipulated science [20-23], used third parties to promote its agenda [24-28], targeted vulnerable populations [29,30], and interfered with regulatory and public policy processes [31-36]. These behaviors are not unique to the tobacco industry; research on internal asbestos and chemical industry documents has uncovered similar actions [37,38]. These similarities suggest that public health researchers can identify patterns of corporate activity by studying tobacco industry documents [16]. The case study presented here highlights the role of inter-company cooperation in advancing global corporate interests, and the power asymmetry between governments and corporations in struggles to regulate public health.

## Methods

Litigation against the tobacco industry has resulted in the public release of over 47 million pages of internal industry documents housed in paper depositories and online electronic archives. The third author first collected documents in 1999 from the paper depository in Minnesota USA, using a computerized index and hand searches to identify documents of interest. From October 2006-March 2007, the first and second authors conducted more comprehensive searches of the online Legacy Tobacco Documents Library [39], the British American Tobacco Documents Archive [40], tobacco company websites [41-43], and other available online collections [44]. (The British American Tobacco Documents Archive was incomplete at the time of our search.) These searches were conducted using snowball sampling, beginning with names of organizations of interest ("ICOSI," "INFOTAB") and using retrieved documents to identify additional search terms. More detailed information on sites and search strategies has been previously published [17,45-48]. Documentary data included letters, meeting minutes, telexes, memos, and reports. We analyzed approximately 1,000 documents to reconstruct the chronology of the organizations and identify their specific foci. Although we outline many of the organizations' activities, given tobacco companies' history of document destruction [49,50], our findings most likely represent a conservative account of their true scope and scale.

## Results

### ICOSI

The first international cross-company issues management organization was established by the chief executive officers of the tobacco companies Philip Morris (PM), British American Tobacco (BAT), R.J. Reynolds (RJR), Reemtsma, Rothmans International, Gallaher, and Imperial Tobacco (UK) in 1977–1978. Named the International Committee on Smoking Issues (ICOSI), its initial purpose was to "establish an agreed industry position on issues of common interest" [51]. Topics of interest to ICOSI were "all those which threaten its freedom of action and which affect the long-term interests of the tobacco industry primarily in the area of smoking and health" (underlining in original) [52]. ICOSI members endorsed an official global tobacco industry position that a "controversy" about smoking and health existed and that additional research was needed to establish whether smoking caused disease [53]. Further, they agreed to "hold the line on admissions concerning what they would admit to their individual governments concerning smoking and health" [[54], p. 189]. As part of that agreement, they pledged to "strenuously" resist government imposition of cigarette warning labels that implied that smoking caused disease, and to avoid making health claims in their advertising [53]. ICOSI incorporated in Switzerland and established an office in Brussels in 1979 [55]. While not a secret organization [56], ICOSI was "a low-key operation" that would not adopt a public role, partly to avoid negative publicity [57,58], and partly to avoid attention from "anti-trust enforcing bodies" [59]. (See Francey and Chapman for additional discussion of ICOSI) [15].

Topping ICOSI's hierarchy was a Board of Directors (composed of two representatives of each founding company, one of whom was the chief executive) which created policy, in part by assembling working parties focused on specific issues [60]. In addition, a secretary general oversaw an information service, intelligence-gathering about tobacco control organizations, and the implementation of ICOSI programs by national manufacturers' associations (NMAs), which played a key role in ICOSI [61,62]. NMAs were perceived as providing a "buffer" to tobacco companies "between controversy and [specific] brands" as well as a "neutral ground" where companies could manage smoking issues [63,64]. More specifically, NMAs acted as ICOSI's local and regional "eyes and ears" and the conduits through which ICOSI policies were enacted and information distributed [58]. In February 1978, there were approximately 9 NMAs in Europe and North and South America [65]; to better protect the industry's interests, ICOSI planned to create a larger NMA network [55].

### Initial ICOSI working groups

ICOSI initially established three working groups. The Smoking Behaviour Working Party was disbanded after only one meeting over concerns that the results of proposed studies on the benefits of smoking could be problematic legally, as they might be interpreted as encouraging people to smoke [66]. The Medical Research Working Party experienced internal conflict [15]. It also appeared to generate hostility among ICOSI board members due to its critical reviews of several ICOSI position papers as biased and inaccurate [67-70], and its view of ICOSI's intention to only pursue research whose "results would prove favourable to the industry" as "unethical" and "downright stupid" [71,72]. It was disbanded by ICOSI's board in September 1979 [73].

The Social Acceptability Working Party (SAWP) was the most long-lived and productive of ICOSI's initial working groups (see Table 1). Its focus was "the level of acceptance of cigarette smoking in society" [74]; its first report outlined the declining social acceptability of smoking in several countries [75]. To combat this, SAWP recommended that the industry focus on secondhand smoke, for "[u]ntil society believes that smoking does not harm the health of nearby nonsmokers, the industry will continue to run grave risks of further reverses" (underlining in original) [75]. SAWP also reported that tobacco control efforts had become highly organized and internationalized through such agencies as the World Health Organization (WHO); these efforts might spread to nations with no negative smoking attitudes [75]. SAWP urged ICOSI to develop countermeasures aimed at blocking government action and influencing public opinion [75].

**Table 1:** Social Acceptability Working Party Projects, 1978–1981

**Project Title, Year(s)**	**Description**	**Outcome(s)**
Public smoking position paper, 1978	44 page paper (drafted by US law firm) arguing secondhand smoke is not harmful to nonsmokers and regulation is unnecessary [62].	• Ratified by member companies, distributed to NMAs [263, 264]. • Updated regularly [136].

11 nation public opinion survey, 1978	Eleven country survey of attitudes on the social acceptability of smoking [265].	• Results presented at NMA workshop in 1979 [266]. • Data, analyses distributed to NMAs [266].

NMA workshops, 1979–1991	Meetings for NMA representatives to exchange information and strategies [75, 267].	Offered yearly [268, 269].

Social costs/social values study, 1978–1981	A project to: • provide NMAS with arguments to counter WHO's assertion that smoking imposed a social cost on society [270]. • document social benefits of smoking [270]. • "drive a wedge" between "anti" and non-smokers [271].	• May 1981 conference at University of Pennsylvania on cost/benefit analysis of the regulation of consumer products, with 6 of 8 speakers industry consultants [270, 272]; only 22 of 10,000 invitees attended [273]. • Proceedings published in book form [274]. • Training program for NMAs to produce data on social benefits of smoking [270]. • Publication of "The Social Costs of Smoking" in Policy Review [275]. • Development of scientific experts (e.g., Dr. Stephen Littlechild, University of Birmingham, UK) [270, 275].

Fourth World Conference on Smoking and Health Task Force, 1978–1979	Committee to prepare for and monitor conference in order to minimize its impact [59].	• Prepared biographies of speakers and background papers on advertising, public smoking, and smoking and health for NMAs and member companies [276–278]. • Arranged for scientific consultants to attend conference [279]. • Monitored the conference and briefed ICOSI members [279]. • Prepared final conference summary [280].

Third World Working Committee, 1978–1979	Subcommittee of 4^th ^World Conference Task Force on Smoking and Health formed to identify and refute likely accusations by conference participants regarding tobacco and the Third World [281].	• Provided background papers to NMAs [83]. • Commissioned UK Economist Intelligence Unit study on the role of tobacco growing in Third World development [282].

Project Mayfly, 1980–1981	Project to develop template for NMA public relations and communication campaigns to "influence, modify, or change public opinion to [sic] the industry, smokers and smoking" [283–285].	Field trials conducted in Australia and New Zealand considered successful [286, 287].

Space restrictions on smoking, 1980	Project to collect and analyze information on public and work place smoking restrictions to help NMAs defend right to smoke in public [78].	Conducted survey of 14 NMAs; results presented at 1980 workshop [288].

Allies project, 1980	Project to identify potential tobacco industry allies and develop strategies to encourage them to defend industry positions [78, 272].	Due to overlap with areas covered by other working parties (i.e., advertising, developing countries), project reassigned to those groups [288].

SAWP's report formed the basis for ICOSI strategies and broader focus from 1978–1980 (see Table 2). During this time, ICOSI committees and task forces established patterns of activities that characterized the organization and its successors for the next several decades: enlisting third party allies (e.g., European tobacco growers, advertising associations) [58], establishing contacts with governmental and United Nations (UN) representatives [58,76], lobbying UN agencies regarding the economic significance of tobacco [77,78], helping to defeat tobacco control legislation (e.g., a Swiss cigarette advertising ban) [79,80], and promoting preferred industry positions via position papers (e.g., "Arguments to Use Against Claims that Tobacco Smoke Is Harmful," distributed in the Middle East) [81], and selective research (e.g., failing to provide the European Commission with research showing that higher cigarette prices lead to reduced consumption) [82-85]. One activity that ICOSI hesitated to engage in was the creation of a voluntary industry marketing code. Advertising was theoretically outside ICOSI's purview as it dealt with commercial issues that had "possible anti-trust implications" [86,87]; thus, early requests to develop such a code to demonstrate the tobacco industry's social responsibility were denied [88-90]. In later years, industry associations overseen by ICOSI's successor organization created voluntary advertising codes "to forestall ... more dramatic bans" in the United Arab Emirates and West Africa [91-95].

**Table 2:** ICOSI committees and task forces, 1978–1981

**Name, year(s)**	**Goal(s)**	**Outcome(s)**
European Economic Community (EEC) Consumerism Task Force, 1978–1980	To prevent the European Commission and European Parliament from enacting legislation restricting cigarette marketing [289].	• Submitted two papers to EEC demonstrating that proposal to ban tobacco advertising would not reduce smoking, and questioning link between smoking and disease [82]. • Mobilized allies (European Trade Union Committee of Food and Allied Workers, tobacco farmers' association, advertising associations) [58]. • Established contacts with representatives of EEC institutions [58]. • Commissioned UK firm (METRA) to analyze industry data to determine the relationship between advertising expenditure and tobacco consumption 1958–1978; it found no significant relationship [84]. When METRA refused to "abandon" its finding that higher cigarette prices led to reduced consumption, ICOSI decided not to provide the European Commission with these results, as they might lead some governments to raise prices [82–85]. • EEC did not enact legislation [290].

Developing Countries Group, 1980–1981	To: • guide ICOSI's response to attacks on tobacco industry's activities in developing countries • work with NMAs to prevent or delay implementation of WHO recommendations to discourage smoking in developing countries • create new NMAs, and encourage them to mobilize tobacco growers in their countries • create allies • address deforestation [103, 291].	• Monitored "international bodies," WHO regional offices, and International Union Against Cancer (UICC) workshops in Venezuela and Argentina [76]. • Helped arrange for two speakers at Venezuela UICC workshop to present industry's view on advertising [76]. • Distributed ICOSI paper "The Threat to the Future of Tobacco Growing and Manufacturing Industry in Developing Countries" to member company affiliates in developing countries [76]. • With help of Council of Malaysian Tobacco Manufacturers, created a model for qualitative research on perceived benefits of smoking, public views of tobacco control movement, and situations where smoking was accepted or not, in order to offer evidence refuting the need for smoking restrictions [76, 292, 293]. • Established personal contacts with Food and Agriculture Organization (FAO) officials [76]. • "Leaf Tobacco: Its Contributions to the Economic and Social Development of the Third World," written by public relations firm Hill and Knowlton and published by Economist Intelligence Unit, made available to NMAs and member companies and distributed to journalists, academic journals, FAO, UN Development Program and UN Center on Transnational Corporation officials; condensed version translated into Spanish [76, 293]. • Indirectly lobbied UN agencies, FAO officials regarding the economic significance of tobacco [77, 78, 117, 294]. • Held regional workshops in Asia and Latin America [132]. • Commissioned economic impact model for developing countries [see Additional file 1] [293].

Effects of Advertising Working Party/Defence of Advertising Committee, 1979–1981	To: • refute argument that advertising induces people to start smoking or smoke more • to demonstrate benefits of cigarette advertising [83, 283].	• Commissioned study of effects of advertising bans on tobacco consumption in Scandinavia which found that price increases and health campaigns had direct (negative) effect on consumption; results not published [283, 289, 295]. • Distributed to NMAs white paper outlining industry's view on advertising, action pack listing material available from ICOSI, and planning guide on how to use the material [283]. • Presented program "Campaign Against Tobacco Advertising Censorship" to NMA workshop [138].

Middle East Working Group, 1980–1981	To defend industry interests in the region [272].	• Drafted voluntary agreement with Kuwaiti government on warning labels and tar and nicotine limits [296, 297]. • Lobbied Iraqi officials regarding warning labels [298]. • Established contacts with Egyptian member of Parliament [299]. • Shook, Hardy and Bacon prepared background briefing papers for use with local agents and distributors ("Arguments to Use Against Claims that Tobacco Smoke is Harmful," "The Smoking and Health Controversy: A Perspective," "Smoking and the Nonsmoker," "Advertising Restrictions Unlikely to Reduce Cigarette Consumption," and "Many Unanswered Questions on Smoking and Health Controversy") [81]. • Wrote media article encouraging health ministers to conduct research "into such areas as might occupy their time for a considerable period" [300, 301].

Product Liability Working Party, 1979	To: • determine position of EEC countries on product liability • examine EEC draft directive on product liability and determine how to change it [302].	• Disbanded as of September 1979 [73].

Swiss Referendum Task Force, 1978–1979	To defeat Swiss referendum to ban all advertising and promotion of tobacco and alcohol [79].	• Helped Swiss NMA develop arguments to oppose the referendum [80]. • Referendum defeated by 59% of Swiss voters in 1979 [58].

Public Position Working Party, 1980–1981	To develop strategies to improve industry credibility [303].	Disbanded after concluding that group's goals overlapped with those of other working groups [304].

### Growing pains

In 1980, ICOSI underwent a series of organizational changes. Gallaher withdrew, citing the time commitment [96]. The Board of Directors chose not to renew the first secretary general's contract when it expired in April 1980. (In a 1998 deposition, PM's Richard Corner indicated, without elaboration, that the reason for termination was "misuse of funds") [[97], p. 20]. Member companies debated whether ICOSI would act simply as a clearing house for tobacco-related information – "a glorified post office" [98] – or whether it would "do or promote its own research and propaganda" [99]. Imperial, concerned about weakening its defenses in future product liability cases, questioned the wisdom of producing position papers "which suggest [ed] industry positions on subjects relating to smoking and health" [100].

Another concern was the failure of NMAs, especially in developing countries, to address long-term threats [101]. Some NMAs worried that taking preventive action on issues that had not yet "registered" locally with the media or public, such as environmental tobacco smoke (ETS, the industry's preferred term to describe secondhand smoke), might draw unwanted attention [102]; others apparently did not understand the threat posed by the globalizing tobacco control movement [101]. Moreover, due to competitiveness between manufacturers, lack of resources, or lack of guidance from senior member company executives to their local-level representatives, NMAs sometimes failed to follow ICOSI policies [103,104].

At meetings in 1980, amidst growing concern about the WHO and the "startlingly" rapid growth of "coordinated anti-smoking activities" among international organizations and intergovernmental agencies, particularly in developing countries, ICOSI members renewed their commitment to a comprehensive, global vision of the organization [13,105]. With WHO preparing an international "attack" on the industry, PM's Jules Hartogh advised that " [i]f we are to stay in the game...we ... must ... develop a worldwide strategy with related actions" [106]. ICOSI would not simply be a clearing house, but would also initiate research and offer analyses to NMAs; create new NMAs; mobilize tobacco growers; seek third party support; and establish directly or indirectly contacts with international organizations (most likely the WHO) [107]. Its information service would also expand [108,109]. Imperial agreed that position papers could be produced under ICOSI's letterhead provided that a disclaimer was added that "the views expressed are not necessarily those of the member companies" [110].

Board members chose a new secretary general (Mary Covington, vice president of PM International's corporate affairs department) [109], and a more "neutral" name for the organization, INFOTAB (drawn from the French translation of the full name, Centre International d'Information du Tabac, or International Tobacco Information Center) [109]. In an apparent effort to emulate the structure of the WHO, whose regional offices "cover [ed] the world," senior ICOSI staff became responsible for servicing NMAs in specific regions [107]. ICOSI's financing also changed: rather than simply dividing all ICOSI costs equally, the companies agreed to share the operating costs equally, but pay for project costs according to market share [109].

### INFOTAB

Throughout 1981 and 1982, INFOTAB was restructured. The Board of Directors disbanded the working groups, replacing them with an advisory group, headed by the secretary general and reporting to the Board, which set policy and appointed ad hoc project teams [111,112]. The secretariat grew, adding a regional coordinator for the Middle East and Africa [113], and an assistant secretary general who was also regional coordinator for Asia [114]. INFOTAB also expanded its membership to include, by invitation, associate members (private enterprises that manufactured tobacco products) and allied members (NMAs, state owned tobacco companies, and private enterprises that either manufactured tobacco products other than cigarettes or provided goods/services to the industry) [115]. By 1984, in addition to its 6 founding members, INFOTAB had 4 associate and 36 allied members, including NMAs in 28 countries and 8 tobacco leaf dealers [116]. It also had 29 "lead companies," overseas subsidiaries or affiliates of a founding company that acted as INFOTAB's eyes and ears in countries without NMAs [117,118]. This membership extended INFOTAB's global reach to 57 countries (see Table 3).

**Table 3:** Countries covered by INFOTAB's network, 1985 [117]

Argentina	Malta
Australia	Mauritius
Bangladesh	Mexico
Barbados	Netherlands
Belgium	New Zealand
Brazil	Nicaragua
Canada	Nigeria
Chile	Norway
Costa Rica	Pakistan
Cyprus	Panama
Denmark	Philippines
Ecuador	Sierra Leone
El Salvador	Singapore
Fiji	South Africa
Finland	Spain
France	Sri Lanka
Germany	Surinam
Ghana	Sweden
Greece	Switzerland
Guatemala	Trinidad
Guyana	Uganda
Honduras	United Kingdom
Hong Kong	United States
India	Uruguay
Ireland	Venezuela
Jamaica	Zaire
Kenya	Zimbabwe
Malawi	Zambia
Malaysia	

As INFOTAB grew, its information services division expanded [119]. Staff produced and regularly updated the "Smoking Issues Status Book," which detailed global smoking legislation and restrictions [114]. They also disseminated summaries of published smoking-related articles [111], case studies of industry actions, reports on tobacco control events, analyses of smoking issues, and reference guides to help members counter allegations about smoking-related diseases and the economic costs of tobacco [119,120]. Information services relied on NMAs, member companies, and consultants to act as its global "intelligence network" and "early warning" system for regulatory threats [121-123].

INFOTAB's information services also maintained a library, conducted research for members, and distributed white papers, action kits, and audio-visual material [124,125]. From 1982–1984, NMAs and member companies used INFOTAB material to argue against advertising restrictions (Argentina and Australia), public smoking bans (Malaysia, Norway), cigarette tax increases (Argentina, Uruguay), and airline and workplace smoking bans (Finland and New Zealand, respectively), and to argue for the economic value of tobacco growing (Panama, Malaysia, Zimbabwe, South Africa, Hong Kong, Australia, and Papua New Guinea) [64,124,126-128]. Much of this material resulted from projects overseen by the advisory group (see Additional file 1).

Other INFOTAB activities included lobbying (via consultants) governmental organizations (e.g., United Nations (UN) Food and Agriculture Organization (FAO)) and government officials (e.g., in the Middle East) [129,130], monitoring tobacco control organizations ([125], p. 4, [131], p. 17), and working through allies, such as the International Union of Advertisers' Associations, which agreed to coordinate with INFOTAB in order to "speak with one voice on all matters related to advertising" (e.g., in opposition to cigarette advertising bans) [132]. INFOTAB also continued to establish new NMAs (Nigeria, Venezuela, and Pakistan), and strengthen existing ones (Argentina) through yearly workshops [133,134].

PM's American law firm Shook, Hardy, and Bacon (SHB) – represented primarily by Don Hoel – played a key role in INFOTAB. PM recommended that INFOTAB hire SHB because PM considered the firm, with its "thorough knowledge of U.S. legal implications," to be the only one capable of providing adequate legal assistance to INFOTAB [135]. To protect members from legal challenges, Hoel attended INFOTAB board meetings and cleared draft meeting minutes, briefing materials, and public relations strategies related to smoking and disease [136]. SHB lawyers monitored international conferences and regularly updated INFOTAB's white paper on public smoking which argued that ETS posed no health risk and that regulation was unnecessary [136,137]. SHB also trained INFOTAB's information services staff regarding information to be stored in the computer (publicly available information rather than "sensitive" internal documents) and how to write abstracts (summaries containing "no judgmental materials") [138].

In 1984, INFOTAB's Board of Directors again reexamined the organization's role and structure [139]. They agreed to "support a more pro-active stance," allowing the secretary general to present industry positions directly to organizations such as WHO and the UN [140]. Board members also expressed tentative support for a higher profile, industry spokesperson role for INFOTAB [140]. Concurrently, INFOTAB scaled back direct involvement in projects, leaving most to NMAs and member companies [141]. INFOTAB's primary focus was now providing information and advisory services and, when necessary, helping coordinate projects. The advisory group was dissolved, and each founding company appointed an INFOTAB liaison [141].

For several years, INFOTAB continued to offer services to NMAs, including annual regional and international information-sharing workshops and a spokespersons' training seminar [142,143]. It also organized (via NMAs, growers, and leaf dealers) lobbying of UN ambassadors in developing nations to oppose WHO's 1986 "Tobacco or Health" Resolution, which called for "a global public health approach and action now to combat the tobacco pandemic" [142,144,145]. Existing projects continued, including an economic impact study of tobacco in Europe designed to counter WHO arguments regarding the high social and economic costs of tobacco by demonstrating the tobacco industry's contributions to the European economy (see Additional file 1) [126,134,146].

But INFOTAB did not take a more public, pro-active posture. INFOTAB's secretary general described INFOTAB as operating in a "reduced role" in a January 1986 memo [147]; one month later, PM's RW Murray indicated that his company wanted INFOTAB to "assume a more pro-active role" [148]. Soon after, INFOTAB established a Global Issues Working Party (GIWP) to "develop a strategic approach to pro-active activities by INFOTAB" [149]. One result of GIWP's efforts was the "Seizing the Initiative" ETS action kit. Its aim was to help NMAs "establish both a credibility and acceptance of balanced scientific evidence presented by the industry" on ETS [150], evidence that supported the industry view that ETS represented an insignificant health risk, a position at odds with regulatory agencies and non-industry funded published research [23,151]. INFOTAB also sought board approval to coordinate a global ETS campaign [152,153], but members expressed doubts about INFOTAB's capabilities. At the US Tobacco Institute, according to an RJR memo, "there is a general feeling that InfoTab [sic] cannot perform on the ETS plan" [154]. Similarly, Brown and Williamson personnel reported that "Infotab is a lot of talk and no action" [155]. In 1988, PM established its own ETS program, Project Whitecoat, and invited other companies to participate [156,157]. Project Whitecoat used third party scientific consultants to disseminate the industry's ETS arguments, successfully delaying or diluting smokefree legislation in Europe, Asia, and Latin America [158-162].

### Barriers to action

One roadblock to effective INFOTAB action was the US legal situation. Under no-fault liability law, tobacco manufacturers could be sued for a defective product that caused harm to consumers, regardless of proof of negligence [163]. In their defense, US tobacco companies typically disputed that there was a causal relationship between smoking and disease, and simultaneously argued that consumers voluntarily assumed the known risks associated with smoking [164] (a stance Philip Morris still maintains in court, even as it claims on its website to agree that smoking causes disease) [165]. The US industry thereby maintained what BAT lawyer Alec Morini deemed a "tightrope policy," in which "no US manufacturer can say that smoking is bad for you, but equally they cannot say that smoking is good for you" [164]. As SHB's Don Hoel reportedly explained at a 1981 INFOTAB Board of Directors meeting, the "U.S. product liability position has to be maintained and extended beyond the U.S. (even where there is no local product liability threat)" [138].

INFOTAB members operating outside the US regarded the tightrope policy as overly "rigid," since it made it "impossible, or at least very difficult for them to act against the anti-smoking propaganda" [166] by, for example, conducting "smoke in moderation" campaigns (which implied that "excessive" smoking was harmful), or by touting the purported health benefits of low tar cigarettes or of smoking in general [164,167]. NMAs called for "more assertive, pro-active activity by the tobacco industry" [168]; however, "the need for caution regarding the primary health issue" sometimes led to inertia [169].

This caution was evident when preparing INFOTAB position papers. In 1980, an RJR lawyer expressed concern that a public smoking paper could be mishandled by "well meaning but inexperienced" NMAs [170]. An incident in the Netherlands was illustrative:

2 officials of the Belgian NMA [were] quoted ... in the leading daily newspaper in the Netherlands as saying that "Two or three packs of cigarettes a day is irresponsible for health and pregnant women should be prudent. ... It is unacceptable to print 'Tobacco causes cancer' on a pack of cigarettes, as asked by the EEC. The cause/effect link has never been scientifically established. ... 'Abuse of tobacco may increase the risk of cancer' is a better warning because this has been proven" [171].

Reporting this incident, SHB lawyer Steve Parrish indicated that " [t]he speakers ... now understand that they were in error, but I do not believe that they understand exactly why they were in error" [171]. Their error may have been condemning *excessive *smoking as irresponsible, thereby implicitly promoting "moderate" smoking. PM and BAT had long recognized the legal dangers of such a theme, as the industry's endorsement of a "healthy" level of smoking could ultimately be used against tobacco companies by plaintiffs who smoked at this level but nonetheless developed diseases [166,172]. A second source of the NMA officials' error may have been stating that it had been *proven *that smoking *might *increase the risk of cancer, wording at odds with the INFOTAB position that there was a "controversy" about whether smoking caused disease that could only be settled by further research [53].

Another factor inhibiting INFOTAB action was inter-company competition. Divergent commercial interests could lead to a lack of consensus on how to manage threats [173]. INFOTAB documents occasionally admonished companies to put aside their differences, as "there are times when possible competitive, short-term gains must be sacrificed to united industry action on smoking issues, in order to achieve longer-term, bottom-line gains for the industry as a whole" [89]. One notable area of conflict was BAT's Barclay cigarette. Barclay was an ultra low-tar cigarette with a filter that produced low machine-measured tar levels, but which was easily compromised by smokers (resulting in higher actual tar deliveries) [174,175]. Upon its introduction, BAT's competitors, particularly PM, engaged in several anti-Barclay activities with various regulatory agencies and government officials. This led to a "paralysis of intra-Industry activities," inhibiting INFOTAB policy development [176-178].

### Refashioning INFOTAB

In 1987, in response to PM's Barclay-related actions, BAT announced its intention to withdraw from INFOTAB [179]. Despite the criticisms leveled at INFOTAB by member companies, leaving the organization was a risky move for BAT. BAT's public affairs manager Robert Ely cautioned that doing so would weaken the company's ability to defend and expand its global markets: BAT would be excluded from its competitors' negotiations with national or regional governments, and a fractured industry would have difficulty fighting tobacco control measures [180]. BAT's subsidiaries also objected to withdrawal, pointing out that INFOTAB was a vital source of information, guidance, and "solidarity against the anti-smoking forces" [181].

BAT's membership expired in May 1990 [179]; PM chose to withdraw from INFOTAB soon after, for reasons that are unclear [182]. An RJR memo suggests that PM's reasons may have included the expense (PM was scheduled to pay nearly half of INFOTAB's proposed £2.5 million 1992 budget) [180,183], INFOTAB's unwieldy bureaucracy, dissatisfaction with delegating industry policy-making to INFOTAB, and a decline in INFOTAB's perceived effectiveness due to lack of involvement of top management, with their "transcending power" to make policy commitments [184]. It was also likely that PM no longer needed INFOTAB; according to BAT, PM had built up a large public affairs department that included two major information centers based in the US and Europe [185]. It had also established a network of six regional corporate affairs divisions dedicated to issues management [186].

According to David Bacon, head of BAT's public affairs department, without PM's funding, INFOTAB could not survive, so "the concept of a 'super global' industry association, responsible for the direction of issues management was finally laid to rest" [187]. In October 1991, the board dissolved the organization (effective, January 1, 1992) [188,189]; it was succeeded by two smaller organizations, the Tobacco Documentation Centre (TDC) and Agro-Tobacco Services (ATS).

### Tobacco Documentation Centre

TDC was founded in 1992 by PM, BAT, RJR, Rothmans, Gallaher and Reemtsma [190]. In 1997, its name was changed to the International Tobacco Documentation Centre, although it continues to use the acronym TDC [191]. It was run by former INFOTAB staff and housed in the former INFOTAB offices in London (INFOTAB had moved into these offices, which were "somewhat difficult to find ... by design" [192] in 1988) [193,194]. But for BAT and PM, TDC was not simply a new INFOTAB. They favored "a very clear and simple definition" of TDC as "an information gathering and dissemination outfit" [193], rather than returning to "business as usual" with a scaled-down INFOTAB, which would send "the wrong signals ...both to the outside world and internally" [195]. BAT's desire to send the right "signals" may have reflected conspiracy charges being leveled at its American subsidiary, Brown and Williamson (BW), in five pending lawsuits in Texas [196]. A "Conspiracy Notebook" assembled by BW/BAT legal consultants noted that INFOTAB might be cited by plaintiffs as evidence that the industry acted in concert to deceive the public about the dangers of smoking [196].

TDC's functions, therefore, were to be limited to collecting and distributing to members publicly available tobacco-related information [190]. BAT (and most likely other founding companies) regarded this as a valuable service because its own information system had been "deliberately curtailed" in order to avoid duplicating INFOTAB's efforts [185]. TDC's charter stated that " [u]nless previously agreed by Charter Members, specifically excluded [from TDC's functions] will be the creation and issue of any original documentation which might be taken to represent an overall industry position" [190]. TDC was barred from engaging in the following INFOTAB activities: "preparation and dissemination of [its own] 'centrally cleared' argumentation," offering "crisis management back-up," organizing industry workshops, forecasting industry-related developments, and taking "a public stance on behalf of the industry" [197,198]. Its initial budget was £1 million, furnished primarily by the founding members [199], and membership was open to NMAs, suppliers, and other tobacco companies [200].

TDC continued INFOTAB's information services, distributing numerous publications, including monthly compilations of global tobacco news, weekly summaries of legislative and media issues, and weekly news printouts [201,202]. Staff also updated the Smoking Issues Status Book [202]. In 1992, TDC distributed to NMAs and lead companies talking points on the US Environmental Protection Agency's draft risk assessment categorizing ETS as a class A carcinogen and background papers on ETS (e.g., "Environmental Tobacco Smoke: Science or Politics?") prepared by a cross-company ETS management group [203,204]. PM's director of corporate affairs Matt Winokur pointed out to PM's chief executive that " [t]his coordinated approach to communications is highly desirable. It enables the entire industry to espouse a common position immediately, an essential element in quickly responding to local government and media" [205] – a statement that might easily have been made about INFOTAB several years earlier.

Between 1996–1998, TDC also hosted several workshops, despite its charter barring this activity. Topics included "assessing the value and quality of published commercial information on the international tobacco business," using the internet to collect tobacco information, and information sources on the tobacco control network [206,207].

The industry intended TDC to have a low external profile. A 1996 RJR document containing employee "media tips" described TDC as an "excellent" information source, but cautioned that "the TDC is not...equipped to handle calls from news reporters or others outside the industry and should not be cited as a source of information" [208]. Instead, TDC "should be cited as information from 'industry estimates' or 'an industry trade group"'[208].

TDC received some media scrutiny in 2001, when aides to US Representative Henry Waxman circulated letters (produced via litigation) written by Ron Tully, TDC's chief executive from 1992–1997 [209]. Tully claimed that he had engaged in numerous illegal activities at the request of TDC's board, including the destruction of 1 million pages of "damaging" INFOTAB and TDC documents [50,210]. He also accused INFOTAB of violating European and American anti-trust laws on numerous occasions (i.e., by discussing pricing strategies) and TDC of denying membership to certain competitors, in violation of its non-profit status in the UK [50,210,211]. (Tully himself stood accused of financial misconduct by the INFOTAB board) [212,213].

TDC still exists; its footprint is visible (though limited) on the internet. A 2004 Gallaher presentation to the UK House of Commons on excise duties cites TDC as an information source [214], as does an Imperial Tobacco 2006 presentation on the Asian market [215]. TDC is also listed in the British Telecom online phone book [216].

### Agro-Tobacco Services

ATS was established by PM, BAT, RJR, Rothmans, Gallaher and Reemtsma in 1992 to continue INFOTAB's coordination of the International Tobacco Growers Association's (ITGA) lobbying activities ([217], pp. 227, 230, 297). ATS staff consisted solely of INFOTAB's Martin Oldman, who appears to have worked with ITGA since 1988, when INFOTAB undertook the transformation of the "largely ineffectual trade association" (established in 1984) into a powerful agricultural lobby to advance tobacco manufacturers' arguments regarding the economic importance of tobacco, particularly in developing nations ([217], p. 230, 218, 219). Like TDC, ATS was registered in Switzerland for tax purposes, but its office was in the UK, initially in the same building as TDC ([220], p. 354). In addition to funding ATS, at Oldman's urging, three of TDC's founders (PM, RJR, and Rothmans) continued INFOTAB's practice of supplying the majority of ITGA's funding ([217], pp. 170, [303,304,221]).

Between 1992–1995, Oldman "control [led] the international voice of agro-tobacco" on behalf of tobacco manufacturers, providing ITGA with reports on the economic viability of tobacco farming, the lack of tobacco crop alternatives, and the role of tobacco in economic development ([217], pp. 112, 307, 222, 223), and producing ITGA's newsletter, which was sent to NMAs, international agencies, governments and the media [217,224]. He also met with (unspecified) Latin American representatives of the UN, WHO, FAO, and the Economic and Social Committee to "build allies...against anti tobacco initiatives" [225,226]. (A WHO report provides more detail on ITGA/ATS activities during this time) [13].

In 1995, the tobacco companies supporting ITGA decided to progressively eliminate their funding, expecting ITGA members to make up the shortfall ([217], p. 5). It is not known why they decided to eliminate direct funding of ITGA, but references in the available documents to maintaining a "discrete interface between the [ITGA] and manufacturers," and to avoiding action that would "necessitate potentially sensitive 'face-to face' contact between individual companies and the [ITGA]" suggest that tobacco companies wanted to avoid public ties to the ITGA [227]. For reasons that are unclear, the tobacco companies also replaced ATS with UK public relations firm Hallmark Marketing Services [228]. Hallmark personnel continued ATS's work, preparing ITGA position papers and news releases, attending regional grower's meetings, offering media training, recruiting new ITGA members, and launching ITGA's website [229,230].

In 1996, Hallmark was paid 113,500 by PM, RJR, BAT, and Rothmans [231,232]. The companies also agreed to fill the gap in ITGA's budget that year, passing the money through Hallmark in order, "for very obvious and important reasons," to keep the companies' connection with ITGA "discreet" [233,234]. In a March 1996 letter to the head of ITGA, Hallmark's Tom Watson explained that his company would be providing the association with £60,000 in return for "specialist consultant services" regarding how to contact tobacco growers' organizations around the world [235]. Hallmark appeared to still be paying for this service in 1999 [236]. In 2000–2001, the focus of Hallmark's activities on behalf of ITGA was minimizing the impact of the FCTC [237].

### Continuing industry cooperation

The tobacco industry has continued to cooperate via NMAs and ad hoc committees. In 1989, INFOTAB's board of directors established in Brussels a regional NMA, the Confederation of European Community Cigarette Manufacturers (CECCM) to "deal exclusively with tobacco industry issues in the European Community," particularly cigarette taxes, environmental tobacco smoke, and advertising restrictions [238-241]. Its members included representatives of BAT, PM, RJR, Rothmans, Gallaher, and Reemtsma [242]. As of 2005, CECCM continued to operate [243].

Cross-company ad hoc committees include an International ETS Management Committee (IEMC) (established by RJR, PM, BAT, Imperial, Rothmans, Gallaher, and BW in 1991) [244] and an International Ingredients Committee (established by 14 tobacco companies in 1993) [245,246]. At a 1995 meeting, it was agreed that IEMC would develop global ETS messages (promoting "accommodation" of smokers and nonsmokers, asserting a lack of scientific evidence of harm to nonsmokers), which would then be distributed in Europe by a regional manufacturing association [247]. Another inter-industry committee, the International Committee of Legal Counsel [248] appears to have been established in 1992 [249]. At meetings held 2–3 times per year, in-house and external lawyers representing multiple companies discuss country-specific litigation developments and exchange information on topics such as preventing litigation, the impact of developments in the US on international litigation positions, and defending ETS cases [250,251].

Nonetheless, in 1995, RJR, lamenting the loss of INFOTAB's structure for coordinating global policies, considered proposing to PM that the two companies coordinate on global issues [184]. It is not known what became of this idea; however, in the mid to late 1990s the international tobacco industry continued to collaborate "when an urgent problem is identified" [184]. These problems included the potential introduction of plain cigarette packaging in Canada in 1995, which prompted creation of a cross-company "plain pack working group" [252], 1999 European Union Commission proposals on tar, nicotine, and cigarette descriptors, which led PM to try to organise industry-wide agreement on delaying tactics [253-255], and the FCTC, whose marketing provisions led Japan Tobacco International, BAT, and PM to create a joint voluntary international marketing code ([256], p. 355).

## Discussion

ICOSI began as a conspiracy among seven tobacco company chief executives to promote internationally the fiction of a "controversy" regarding smoking and disease [15]. It quickly developed into a multi-million dollar global organization with a new name, expanding membership, and a broader mandate. Relying on a network of centralized staff, member company senior personnel, consultants, lawyers, and NMAs, ICOSI's successor, INFOTAB, operated as an anti-WHO. Its mission was to systematically thwart public health by globalizing "doubt" not only about smoking and disease, but also about the economic costs of tobacco, the social costs of smoking, the motivations of tobacco control advocates, the relationship between smoking and advertising, and the need for smoking restrictions. Where it succeeded, INFOTAB unquestionably facilitated the spread of the global tobacco disease epidemic.

INFOTAB also created and served as the nucleus of a world tobacco community. This community encompassed all stages of the process of transforming tobacco into a commodity – growers, leaf dealers, manufacturers, and advertisers. But cigarette manufacturers and their attorneys played the biggest role. Under their explicit direction, INFOTAB set policies and crafted strategies that ensured that the global tobacco community spoke and acted as one. Such unity protected the tobacco industry as a whole, by discouraging individual companies from engaging in actions – such as compromising with governments – that might negatively affect other companies. Shared policies also disproportionately benefited the most legally vulnerable but economically privileged members of the industry, US tobacco companies, by ensuring that no tobacco manufacturer anywhere in the world directly or indirectly admitted that smoking caused disease. With US lawyers vetting every INFOTAB meeting and "product" (position paper, meeting minutes), the concept of protecting the US industry was deeply ingrained in the organization.

Developing nations were especially vulnerable to INFOTAB's global strategies. Those that grew tobacco were and are the object of sustained lobbying efforts regarding the economic value of the crop. That INFOTAB's founders continued this program after INFOTAB was dissolved suggests that they regarded it as particularly successful. Developing nations were also the focus of INFOTAB's attentions via national and regional manufacturers' associations. These associations may have faced less resistance when implementing INFOTAB sanctioned tactics, as the countries in which they operated had fewer resources with which to challenge them.

Although INFOTAB devolved into two smaller organizations, the global infrastructure and cooperative spirit it created survive. Cross-company committees continue to create common policies, positions, and strategies, and TDC allows for the rapid dissemination of this information among a global network of national and regional manufacturing associations. These associations, in turn, are very active, submitting information to public hearings on the FCTC that repeats arguments initially developed by INFOTAB regarding the "freedom to choose" to smoke and the economic importance of tobacco, particularly in developing nations (an argument also promulgated by the INFOTAB and ATS-backed ITGA) [257-261]. While these associations claim to represent national or regional interests, it is important for policymakers to recognize that they are not independent, but are allied with a larger, worldwide confederation of multinational tobacco companies. The FCTC requires governments to protect tobacco control policies from the "commercial and other vested interests of the tobacco industry" [262]; governments must therefore be alerted that even tobacco companies that have no visible presence in their countries may play a role via these covertly controlled "super global" networks. The US NMA was shut down as part of the 1997 Master Settlement Agreement; a similar remedy could be sought in other countries in order to protect the public from the devastating consequences of systematic industry interference in tobacco control policymaking. All countries should institute policies requiring that researchers, lobbyists, and others representing themselves as stakeholders in tobacco control policy decisions fully disclose any financial and other ties to tobacco companies, NMAs, and/or other affiliates acting on behalf of the tobacco industry's interests in any capacity, and setting strong penalties for failure to do so.

## Conclusion

The massive scale and scope of this industry effort illustrate how corporate interests, when threatened by global public health initiatives, sidestep competitive concerns in order to coordinate their activities. Other international public health movements should look for evidence of similar coordinated behavior on the part of other industries that have global interests in obstructing effective public health policies.

## Competing interests

REM and GI separately each own one share of Altria and Reynolds American stock for research and advocacy purposes. REM and PAM served as tobacco industry documents consultants for the Department of Justice in *United States of America v. Philip Morris et al*.

## Authors' contributions

PAM collected and analyzed data, wrote the first draft of the manuscript, and revised subsequent drafts. GI collected and analyzed data and helped edit the manuscript. REM conceived of the study, collected and analyzed data, participated in its design and coordination, and helped draft and edit the manuscript. All authors read and approved the final manuscript.

## Supplementary Material

Additional File 1 Table 4. INFOTAB projects, 1981–1991. The table describes 29 projects initiated by INFOTAB between 1981 and 1991 and their outcomes. Click here for file
