# Supplementary material for: Tobacco industry issues management organizations: Creating a global corporate network to undermine public health
Source: Global Health. 2008 Jan 17;4:2. doi: 10.1186/1744-8603-4-2 (PMC2265275; doi:10.1186/1744-8603-4-2)
Supplement: Additional File 1 — Table 4. INFOTAB projects, 1981–1991. The table describes 29 projects initiated by INFOTAB between 1981 and 1991 and their outcomes. [file 1744-8603-4-2-S1.pdf]

Table 4. INFOTAB projects, 1981-1991 (page 1)

Project, year(s)	Description	Outcome(s)
Mobilization of leaf sector, 1981-1991	Project to determine how to mobilize tobacco grower and leaf dealer support.[1]	• Surveyed NMAs and member companies to assess use of the Economist Intelligence Unit (EIU) publication “Leaf Tobacco: Its Contributions to the Economic and Social Development of the Third World” when lobbying UN Food and Agriculture Organization (FAO).[2]<br>• Prepared paper “Further Usage of the EIU Leaf Tobacco Study” for distribution to NMAs and member companies.[3]<br>• Prepared paper “Threats to the Future of Tobacco Growing and Manufacturing Industry in Developing Countries” and distributed to NMAs, member companies, and tobacco leaf merchants.[1, 3]<br>• Produced audio-visual program “Target Tobacco” for tobacco growers.[4]<br>• Organized international meeting of leaf dealers in US (1984) to introduce them to INFOTAB and urge cooperation.[5]<br>• Helped transform International Tobacco Growers Association (ITGA) into an organization that lobbied WHO and FAO regarding economic benefits of tobacco growing.[6]
Middle East Working Group, 1981-1986	INFOTAB assumed responsibility for chairing group in 1981.[7] Members (representatives of BAT, BW, PM, RJR, Reemtsma, Gallaher, and Rothmans) met bi-monthly to exchange information and create guidelines for member companies operating in Middle East.[8, 9]	• Monitored regional media, WHO regional office, and International Union Against Cancer (UICC) regional meetings.[10, 11]<br>• Revised and translated into Arabic INFOTAB argumentation on advertising and passive smoking.[10, 12]<br>• Negotiated with Sudanese government on tobacco legislation.[13]<br>• Collected and distributed existing tobacco laws and regulations in Middle East.[12]<br>• Drafted paper on relationship between Islam and smoking.[14]<br>• Agreed to standardized style for warning labels on cigarette ads.[10, 15]<br>• Provided advertising argumentation to Kuwaiti publishers.[11]<br>• Provided protocol for measuring tar and nicotine to laboratories in Saudi Arabia, Kuwait, and Qatar in order to “maintain an uninterrupted flow of product into the market.” [8, 16] and to “ensure the continued maintenance of the highest possible constituent levels.” [17] (Kuwait and Saudi Arabia adopted recommendations.) [18]<br>• Lobbied Gulf Cooperation Council (GCC) countries not to require reduced tar and nicotine yields in cigarettes, and to allow manufacturers to place warning labels on the side of the pack rather than the front.[17]<br>• Group disbanded in 1986 when BAT, BW, and Rothmans resigned over PM’s competitive activities.[19]
Middle East Tobacco Association (META), 1988-2007 (http://www.dubaitrade.ae/portal/clientList.do)	Group (founded by Rothmans, BAT, BW, Gallaher, PM, RJR, and chaired until 1991 by INFOTAB consultant).[20] to “promot[e] and defend the Tobacco Industry in the [Gulf Cooperation Council] countries.”[20]	• Created internal voluntary advertising and promotions code for United Arab Emirates to “forestall … more dramatic bans.” [21, 22] This would be made available to government authorities “as and when necessary.” [23]<br>• Lobbied business owners and health officials to delay or abandon public place smoking restrictions.[24]<br>• Working through United Arab Emirates (UAE) chapter of the International Advertising Association, successfully prevented implementation of proposed UAE ad ban in 1990.[25]<br>• Successfully lobbied non-META tobacco manufacturers to follow META policy of “only comply[ing] with what the law…require[s]” regarding cigarette pack labeling.[26, 27]<br>• Implemented media strategy through Middle East Marketing and Communications (MEMAC) to help delay “implementation of proposals which…would have damaged the industry” and to create “awareness of some of the good of the industry without causing offence to any official.” [28] MEMAC met with editors and placed 83 articles in the regional and Pan Arab press in 1992, 128 in 1993, and 174 in 1994.[23, 29] It also “suppress[ed] anti-smoking material through good relations with the editors” in Qatar.[29]

From: McDaniel PA, Intinarelli G, Malone RE: **Tobacco industry issues management organizations: Creating a global corporate network to undermine public health,** *Globalization and Health*, 2007.

Table 4. INFOTAB projects, 1981-1991 (page 2)

Project, year(s)	Description	Outcome(s)
Middle East Tobacco Association (META), 1988-2007 (http://www.dubaitrade.ae/portal/clientList.do) [**cont'd from previous page**]	Group (founded by Rothmans, BAT, BW, Gallaher, PM, RJR, and chaired until 1991 by INFOTAB consultant).[20] to "promot[e] and defend the Tobacco Industry in the [Gulf Cooperation Council] countries."[20]	• Monitored (through MEMAC) anti-smoking material, conferences, and activities and maintained database on regional "anti smoking personalities." [29]<br>• MEMAC successfully lobbied GCC officials to oppose tobacco ad ban in 1994.[29]<br>• Approved MEMAC "voice of reason" strategy to use Arab writers to develop pro-industry articles.[23]<br>• Created Tax Sub Committee to reach consensus on desired structure of customs duty on cigarettes.[25]<br>• Petitioned Qatar against a proposed advertising ban.[30]<br>• Established Bahrain, Kuwait, Oman, Iran, UAE, Lebanon, and Qatar Working Groups involving META company field managers.[30-35]<br>• Submitted argumentation on ad bans, public place smoking, and tar and nicotine levels to Gulf Anti-Smoking Committee.[23]<br>• Lobbied ministers of health, finance, and commerce to oppose proposed mandatory reduction in tar and nicotine in cigarettes sold in GCC countries.[32, 36] Successful in 1991.[25]<br>• Created Scientists Sub Committee to lobby GCC scientists and standards organization officials against limiting tar and nicotine levels.[25, 37, 38]<br>• Created voluntary marketing code of conduct for Lebanon.[39]<br>• Launched a courtesy campaign – "Check it's all right before you light." [40]<br>• Launched retailer programs in 4 GCC countries to prohibit sales to youth (under 18).[41]<br>• Launched youth smoking prevention program, "Kid's World," aimed at parents, teachers, and government officials.[41]
West Africa Working Group, 1981-1991	Group of representatives of 8 tobacco companies organized to create voluntary advertising codes to pre-empt government restrictions.[42, 43] INFOTAB acted as "neutral chair" of group.[43]	• Coordinated successful industry effort to repeal cigarette advertising ban on Gabonese radio in 1984.[44]<br>• Created voluntary advertising code for use in Ivory Coast and Senegal in 1986, Togo, Niger, and Benin in 1988, Mauritania and Liberia in 1989, Burkina Faso in 1990.[44-53] Helped create/coordinate national working groups in Burkina Faso, Ivory Coast, Guinea, Niger, Cameroon, Nigeria, Uganda, Zaire, and Togo.[54, 55]
Mayfly II, 1982-1983	Project to develop kit for NMAs containing communications handbook, suggestions for how to work with allies and target groups, and catalog of industry case histories.[56]	• Kit produced ("Preparing for Communications," "Act and Communicate on Tobacco," and "The Results of Communication: The Industry in Action").[57]
Economic impact methodology for less developed countries, 1982	A study to develop model to measure economic impact of tobacco industry in developing countries (for use by NMAs or tobacco companies).[58]	• Once model established, economic impact studies conducted in Panama, Malaysia, Zimbabwe, South Africa, Hong Kong, Australia, Papua New Guinea, Brazil, Belgium, Guatemala, N. Ireland, New Zealand, Jamaica, and Canada.[57, 59, 60]<br>• Results of Malaysian study published in brochure and distributed nationally (estimated to have reached 35% of population); as a result, no new taxes imposed.[60, 61] Zimbabwean study distributed to government officials.[61]
Economic impact methodology for industrialized nations,1982	Funding for economist Ingo Walter (New York University) to develop model to measure economic impact of the tobacco industry in industrialized nations.[62]	• Produced and distributed to NMAs the booklet "A Guide to Measurement of the Economic Impact of the Tobacco Industry on the National Economy." [62]<br>• In 1985, produced economic impact study of tobacco industry in Western Europe.[63]

From: McDaniel PA, Intinarelli G, Malone RE: **Tobacco industry issues management organizations: Creating a global corporate network to undermine public health,** *Globalization and Health,* 2007.

Table 4. INFOTAB projects, 1981-1991 (page 3)

Project, year(s)	Description	Outcome(s)
10 nation study, 1981-1982	An update of original 11 nation study (see table 3) to identify "those areas where the tobacco industry is viewed favourably." [64]	• Terminated in 1982 due to doubts about its usefulness.[65, 66]
Social costs/social values (continuation of Social Acceptability Working Party project), 1981-1986	Project to refute the idea that smoking creates social costs.[67]	• Paper published in "Personnel Administrator" arguing that smokers no more costly to employ than non-smokers.[56]<br>• 1985 publication of book "Smoking and Society: Toward a More Balanced Assessment" edited by economist Robert Tollison (George Mason University), with chapters on "Smoking and Health," "Smoking, Human Rights, and Civil Liberties," and "A Sociological View of the Anti-Smoking Phenomenon." [68]<br>• 1986 publication in *Public Choice* of "The Political Economy of the Restriction of Choice" by S. Littlechild and J. Wiseman.[69]
Social role of smoking, 1981-1982	Project to develop public relations program "to reaffirm the smoker and make non-smokers aware of the positive social aspects of smoking to the smoker."[70]	• A method to communicate findings from sociological studies was incorporated into Mayfly II.[1]<br>• Test in Germany of a public forum on smoking, with public health official asserting that secondhand smoke is merely an "annoyance," a judge arguing that courts should not be involved, a sociologist discussing role of smoking in society, and a psychologist discussing interpersonal conflicts.[66, 71]
Identify and brief experts on public smoking in developing countries, 1981	Pilot project in South America to brief scientists on scientific literature on passive smoking, and encourage them to conduct research to strengthen industry's position.[72]	Unknown. (In 1992, PM and BAT initiated "The Latin Project," which had similar goals.) [73]
Smoking and drugs, 1981	Financial support for study to prove smoking unrelated to drug addiction.[1, 74]	Unknown – not published as of 1984.[75]
Anti-smoking motivations, 1982	Financial support for study of German non-smokers to show that "the motivation of anti-smokers is essentially a power drive."[76]	Unknown.
European Economic Community (EEC) Competence, 1981-1982	Sponsorship of symposium on constitutional limits of EEC action on cultural, sports, and health issues.[76]	• Symposium held in 1981.[1]<br>• Proceedings to be published in 1982.[1]
Advertising and smoking, 1981-1991	Project to develop publications to support tobacco advertising.	• Leaflets and booklet ("The Five Arguments against Tobacco Advertising Censorship")[77] presenting case against advertising "censorship" published by International Advertising Association (in English and Spanish) [60]; INFOTAB added four more arguments and distributed nine separate booklets.[59, 78]<br>• Material used in 1982 to convince Argentina's minister of health not to support television ad ban.[79]<br>• Produced kit for NMAs to defend ability to advertise, with campaign planning guide, guidelines for dealing with media, draft speeches, press releases, letters, and presentations.[43]<br>• Arranged publication by the International Advertising Association of three reports by the Children's Research Unit (UK) arguing that advertising plays no role in youth smoking initiation.[80]<br>• Presentation by UK Advertising Association on "Advertising, Brands, and Markets" to Council of Europe.[60]

Table 4. INFOTAB projects, 1981-1991 (page 4)

Project, year(s)	Description	Outcome(s)
Public Smoking Project, 1982	Project to help NMAs respond to restrictions on smoking in public places.[81]	• Asked US Tobacco Institute to complete project.[76]
Taxation, 1984	Development of kit for NMAs containing counter-arguments, research, and action to prevent tax increases on cigarettes.[82]	• Kits distributed to NMAs [83] containing "Consumer Action on Taxation," a blueprint for NMAs to "politicise the taxation issue to their advantage" via petition drive against tobacco tax.[84]<br>• Video of presentation by Professor Ingo Walter on "Cigarette Taxation" distributed to NMAs to help fight tax increases.[4, 57]
Public smoking film ("Beyond Reasonable Doubt"), 1983	PM film on secondhand smoke, translated into French, Spanish, and Portuguese.[85, 86]	• Made available to NMAs.[87]<br>• Prepared brochure to accompany film in 1984.[75]
5th World Conference on Smoking or Health, 1982-1983	Team to monitor and report on conference, arrange for industry-friendly experts to attend, and distribute papers to refute conference arguments.	• Mailing to NMAs of briefing papers covering key issues likely to be raised at conference.[88]<br>• Daily reports from 16 monitors on conference sessions, including audience numbers, identification of "more vocal members of the audience," and audience reaction.[89]<br>• Final report detailing "anti-tobacco blueprint" for action.[90]<br>• Audiotapes of key conference presentations sent to NMAs.[59]
Passive Smoking Assessment Group, 1984	Team to recommend how to improve the industry's scientific position on passive smoking and the political climate.[91, 92]	• Recommended INFOTAB work with UK Tobacco Advisory Committee and German NMA to coordinate "productive exchanges" of scientific work; also suggested INFOTAB consider grants for scientific research.[93, 94]<br>• In 1987, INFOTAB created environmental tobacco smoke (ETS) kitset of public affairs materials for NMAs to use to create a campaign to "maintain doubt on the scientific front about the effects of ETS." [95-97]
Developing Countries Strategy Group, 1983-?	Team to assess and improve and ability of NMAs in developing countries to pre-empt or counter attacks on the industry by international organizations such as the UN; to enlist government officials as allies; to gain allies in countries where the industry had no established presence; and address deforestation.[60, 98]	• Lobbied (via former US secretary of agriculture Orville Freeman) delegates of Rome FAO conference in 1983 about economic importance of tobacco.[4, 99]<br>• Gave slide presentation on how to thwart global attacks on tobacco industry to agricultural ministers in Indonesia, Zambia and Tanzania in 1984,[60, 75, 82, 100] and to UN ambassadors of Nigeria, Mexico, and Argentina.[57, 63]<br>• Briefed UN ambassadors and FAO representatives prior to 1989 World Health Assembly on economic importance of tobacco in developing countries.[101]<br>• Commissioned International Forest Science Consultancy to "show the negligible effect of tobacco curing on deforestation."[63, 102]
Economic Impact of Tobacco in European Community (EC), 1984-1985	Study of economic impact of tobacco industry in Western Europe to counterbalance WHO reports regarding the high social and economic costs of tobacco.[60, 103, 104]}	3,000 copies of study (in 6 languages) prepared; presented to EC Commission directorates and economists.[105]
Courtesy campaign, 1984	Development of kit for NMAs on courteous public smoking campaign.[82]	• Tested in UK in 1984; initial results unsatisfactory due to "perceived elements of pressure on smokers."[106]<br>• Further development led to suggestions for NMAs on how to create a courtesy campaign that prevented "tolerant" nonsmokers from becoming "anti-smokers."[107]

Table 4. INFOTAB projects, 1981-1991 (page 5)

Project, year(s)	Description	Outcome(s)
Global Issues Working Party, 1986-?	Group to develop strategic approach to pro-active activities by INFOTAB in 6 areas: World Conference on Tobacco or Health, EEC, Taxation, ETS, World Health Assembly, and labeling.[108]	• Developed “seizing the initiative” ETS action kit to help NMAs “establish both a credibility and acceptance of balanced scientific evidence presented by the industry on the issue of ETS.”[109] • Briefed UN ambassadors on economic importance of tobacco.[110]
6th World Conference on Tobacco or Health, 1987	Team to monitor and report on conference, distribute press releases and briefing papers to refute conference arguments, and issue daily conference reports to industry.[111, 112]	Distributed daily summaries, and synopsis and implications of conference to NMAs and member companies.[113-115]
International Public Affairs Committee, 1989-1991	Unknown – limited documentation available.[116, 117]	Unknown.
International Agriculture Committee, 1989-?	Group of tobacco leaf dealers and processors organized by INFOTAB to • organize funding and strategize on behalf of agriculture lobby • supervise ITGA publications.[118, 119]	Unknown – limited documentation available.
7th World Conference on Tobacco or Health, 1990	Team to monitor and report on conference, and distribute papers to refute conference arguments.	• Provided background material to NMAs,[120] including media-ready material on the importance of the world tobacco trade.[121] • Issued daily monitoring reports.[122]
Global Argumentation Project, 1990-1991	Group to develop papers for NMAs for lobbying and public relations campaigns.[123]	Limited available documents indicate that BAT and PM circulated to INFOTAB members drafts of “claims and responses” material on a variety of smoking issues.[124] Shook, Hardy and Bacon also prepared position papers on pesticides and on “other cancers,” presumably cancers other than lung cancer.[125]

Abbreviations: BAT, British American Tobacco; BW, Brown and Williamson; EEC, European Economic Community; EIU, Economist Intelligence Unit; ETS, environmental tobacco smoke; GCC, Gulf Cooperation Council; ITGA, International Tobacco Growers’ Association; MEMAC, Middle East Marketing and Communications; NMA, national manufacturers’ association; PM, Philip Morris; RJR, RJ Reynolds; UICC, International Union Against Cancer; UAE, United Arab Emirates; UN FAO, United Nations Food and Agriculture Organization; WHO, World Health Organization

From: McDaniel PA, Intinarelli G, Malone RE: **Tobacco industry issues management organizations: Creating a global corporate network to undermine public health,** *Globalization and Health*, 2007.

References

1. INFOTAB. 1982. Item 5 report from the advisory group. Nov. Philip Morris. http://legacy.library.ucsf.edu/tid/dxn04e00.
2. Covington MW. INFOTAB. 1982. Progress report 1st quarter 1982. 23 Apr. British American Tobacco. http://bat.library.ucsf.edu//tid/dfl00a99.
3. Leach MJ. 1982. Project progress report. Motivating and mobilising leaf growers and related groups at the national level. 13 Jul. RJ Reynolds. http://legacy.library.ucsf.edu/tid/pzz19d00.
4. INFOTAB. 1984. Latin American workshop, Doral Hotel, Miami. 21 Mar. Brown & Williamson. http://legacy.library.ucsf.edu/tid/hho01f00.
5. INFOTAB. 1984. Meeting with representatives of international leaf dealers Greenbrier 04 May. Brown & Williamson. http://legacy.library.ucsf.edu/tid/rsf33f00.
6. Bloxcidge JA. INFOTAB. 1988. International Tobacco Growers' Association (ITGA). 11 Oct. British American Tobacco. http://bat.library.ucsf.edu//tid/sik47a99.
7. Borek A. 1981. Meeting of the Middle East Working Group Brussels on 810917. 17 Sep. Philip Morris. http://legacy.library.ucsf.edu/tid/fgr25e00.
8. Stimpson B. INFOTAB. 1985. Middle East Working Group-statement of role and structure. 18 Oct. British American Tobacco. http://bat.library.ucsf.edu//tid/plq00a99.
9. Leach M. 1986. Note from Michael Leach to EAA Bruell regarding INFOTAB. 20 Oct. British American Tobacco. http://bat.library.ucsf.edu//tid/jyh00a99.
10. Middle East Working Group. 1983. Projects. 04 Jul. Brown & Williamson. http://legacy.library.ucsf.edu/tid/mqo93f00.
11. Moore GW. 1983. Minutes of the Middle East Working Group meeting held in Larnaca, Cyprus. 11 Nov. Philip Morris. http://legacy.library.ucsf.edu/tid/cqw46e00.
12. Corner RM. INFOTAB. 1985. Letter regarding project documentation. 23 Nov. British American Tobacco. http://bat.library.ucsf.edu//tid/tkq00a99.
13. Simpson B. INFOTAB. 1983. Item 3. Report from the secretary general. Oct. http://tobaccodocuments.org/rjr/503886951-6986.html.
14. [Covington MW]. 1983. Item 3. Report from the secretary general. Oct. RJ Reynolds. http://legacy.library.ucsf.edu/tid/dvu75d00.
15. Mylonas D. 1983. Middle East Working Group minutes of meeting - 830421. 21 Apr. Brown & Williamson. http://legacy.library.ucsf.edu/tid/cxk02d00.
16. Scott MB. 1984. [I enclose a copy of the draft protocol]. 31 Jan. Brown & Williamson. http://legacy.library.ucsf.edu/tid/fnm90f00.
17. Middle East Working Group. 1986. Draft minutes Middle East Working Group Geneva. 17 Jul. Brown & Williamson. http://legacy.library.ucsf.edu/tid/mbk61f00.
18. Philip Morris. 1989. EFTA, Eastern Europe, Middle East, Africa long range plan 1990-1992. Dec. http://tobaccodocuments.org/pm/2500066142-6294.html.
19. Marcotullio RJ. 1986. INFOTAB board of directors meeting - Hamburg, November 4, 1986. 10 Nov. RJ Reynolds. http://legacy.library.ucsf.edu/tid/sxu82d00.
20. INFOTAB. 1988. Middle East Tobacco Association: the coordinator duties, responsibilities and limitations. 21 Jan. British American Tobacco. http://bat.library.ucsf.edu//tid/ghp16a99.
21. Middle East Tobacco Association. 1991. Voluntary code of cigarette advertising and promotions United Arab Emirates. Philip Morris. http://legacy.library.ucsf.edu/tid/hwe87e00.
22. Middle East Tobacco Association. 1991. Final draft voluntary in-house code of cigarette advertising & promotions United Arab Emirates. 18 Oct. Philip Morris. http://legacy.library.ucsf.edu/tid/zxs25c00.
23. Allen R. Middle East Tobacco Association. 1992. Draft minutes 15th META meeting. 02 Jul. Philip Morris. http://legacy.library.ucsf.edu/tid/kfi81f00.

From: McDaniel PA, Intinarelli G, Malone RE: **Tobacco industry issues management organizations: Creating a global corporate network to undermine public health,** *Globalization and Health,* 2007.

24. Allen R. Middle East Tobacco Association. 1992. Public smoking: the industry and the campaign against public smoking (ETS) in the GCC. 14 Oct. Philip Morris. http://legacy.library.ucsf.edu/tid/cgy85e00.
25. Philip Morris. 1991. [Media strategy]. Sep. http://legacy.library.ucsf.edu/tid/iyg22e00.
26. Borek A. Middle East Tobacco Association. 1989. Labelling requirements for tobacco products in the Arab Gulf States. 29 Mar. British American Tobacco. http://bat.library.ucsf.edu//tid/cdp16a99.
27. Elstgeest K. Agio Sigarenfabrieken. 1989. Letter from Kees Elstgeest to Abdullah Borek regarding tar and nicotine. 03 May. British American Tobacco. http://bat.library.ucsf.edu//tid/bdp16a99.
28. Allen R. Middle East Tobacco Association. 1991. 12th META meeting Geneva October 17/18, 1991. 15 Sep. British American Tobacco. http://bat.library.ucsf.edu//tid/jmc26a99.
29. Davies R. Middle East Tobacco Association. 1995. Middle East: META management summary report October - December 1994. 20 Dec. British American Tobacco. http://bat.library.ucsf.edu//tid/vrb44a99.
30. Allen R. Middle East Tobacco Association. 1992. Amended draft minutes 14th META meeting. 23 Oct. Philip Morris. http://legacy.library.ucsf.edu/tid/fni14e00.
31. Middle East Tobacco Association. 1991. META's priorities and proposed work plan 1992-1993. 17 Dec. Philip Morris. http://legacy.library.ucsf.edu/tid/dpi14e00.
32. Davies R. Middle East Tobacco Association. 1994. SASO meeting. 22 Dec. British American Tobacco. http://bat.library.ucsf.edu//tid/fhn60a99.
33. Hay CNK. Middle East Tobacco Association. 1995. Draft minutes of Iran meeting. 30 Apr. British American Tobacco. http://bat.library.ucsf.edu//tid/yrb44a99.
34. Hay CNK. Middle East Tobacco Association. 1995. UAE Working Group meeting. 05 Jul. British American Tobacco. http://bat.library.ucsf.edu//tid/ntb44a99
35. Hay CNK. Middle East Tobacco Association. 1995. Lebanon meeting. 13 Aug. British American Tobacco. http://bat.library.ucsf.edu//tid/grb44a99.
36. Hay CNK. Middle East Tobacco Association. 1995. Saudi Arabia-MCLs action plan. 22 Sep. British American Tobacco. http://bat.library.ucsf.edu//tid/acg60a99.
37. Allen R. Middle East Tobacco Association. 1990. META scientists and public affairs meeting. 19 Mar. Philip Morris. http://legacy.library.ucsf.edu/tid/xkb29e00.
38. Dymond HFD. 1992. Minutes of META scientists meeting. 13 Apr. Philip Morris. http://legacy.library.ucsf.edu/tid/ify85e00.
39. Hay CNK. Middle East Tobacco Association. 1995. Lebanon voluntary code. 09 Nov. British American Tobacco. http://bat.library.ucsf.edu//tid/arb44a99.
40. Tobacco Merchants Association. 1996. World alert. 29 May. Brown & Williamson. http://legacy.library.ucsf.edu/tid/pcg11c00.
41. Philip Morris. 1999. Draft response "Dar Assayad" - requires review 23 Dec. http://legacy.library.ucsf.edu/tid/bcz32c00.
42. Murphy JFG. 1986. Minutes of executive committee meeting held on 9th December 1986. 11 Dec. British American Tobacco. http://bat.library.ucsf.edu//tid/rnw03a99.
43. Corner RW. 1984. Advertising; freedom of advertising and sponsorship: CATAC - revised arguments. Oct. RJ Reynolds. http://legacy.library.ucsf.edu/tid/kxu75d00.
44. 1984. West Africa Working Group (WAWG). Philip Morris. http://legacy.library.ucsf.edu/tid/gzu71f00.
45. Covington MW. 1982. Item 4 secretariat functional activities. Nov. Philip Morris. http://legacy.library.ucsf.edu/tid/dir25e00.
46. Swan D. Tobacco Advisory C. 1993. Ad Ban - EC & UK Developments: Revised Draft Letter to Secretary of State for Health. 29 Oct. British American Tobacco. http://bat.library.ucsf.edu//tid/qfx16a99.
47. [West Africa Working Group]. 1990. Voluntary code of cigarette advertising in Togo. May. Philip Morris. http://legacy.library.ucsf.edu/tid/abi49e00.

From: McDaniel PA, Intinarelli G, Malone RE: **Tobacco industry issues management organizations: Creating a global corporate network to undermine public health,** *Globalization and Health,* 2007.

48. [West Africa Working Group]. 1990. Voluntary code of cigarette advertising in Benin. May. Philip Morris. http://legacy.library.ucsf.edu/tid/aci49e00.
49. [West Africa Working Group]. 1990. Voluntary code of cigarette advertising in Mauritania. May. Philip Morris. http://legacy.library.ucsf.edu/tid/hbi49e00.
50. [West Africa Working Group]. 1990. Voluntary code of cigarette advertising in Burkina Faso. May. Philip Morris. http://legacy.library.ucsf.edu/tid/jlj22e00.
51. [West Africa Working Group]. 1990. Voluntary code of cigarette advertising in Liberia. May. Philip Morris. http://legacy.library.ucsf.edu/tid/mbi49e00.
52. [West Africa Working Group]. 1990. Voluntary code of cigarette advertising in Senegal. May. Philip Morris. http://legacy.library.ucsf.edu/tid/zai49e00.
53. [West Africa Working Group]. 1990. Voluntary code of cigarette advertising in Niger. May. Philip Morris. http://legacy.library.ucsf.edu/tid/wbi49e00.
54. West Africa Working Group. 1991. Hamburg February 8, 1991 draft minutes. 08 Feb. Philip Morris. http://legacy.library.ucsf.edu/tid/glj22e00.
55. West Africa Working Group. 1991. Athens November 22, 1991 draft minutes. 22 Nov. Philip Morris. http://legacy.library.ucsf.edu/tid/bci49e00.
56. INFOTAB. 1983. Board of directors meeting Brussels. 12 Apr. Philip Morris. http://legacy.library.ucsf.edu/tid/pgp02a00.
57. British American Tobacco. 1986. A general briefing on INFOTAB. Jan. http://bat.library.ucsf.edu//tid/jxa30a99.
58. [Picton TJ]. INFOTAB. 1982. Project progress report. 24 Nov. RJ Reynolds. http://legacy.library.ucsf.edu/tid/kzz19d00.
59. INFOTAB. 1984. INFOTAB board of directors meeting Brussels. 04 Apr. Philip Morris. http://legacy.library.ucsf.edu/tid/bds02a00.
60. INFOTAB. 1984. INFOTAB board of directors meeting Phoenix. 29 Oct. RJ Reynolds. http://legacy.library.ucsf.edu/tid/lkb71d00.
61. INFOTAB. 1984. NMA workshop Brussels - 19841008 - 19841011. 08 Oct. Tobacco Institute. DOJ CIVIL. http://legacy.library.ucsf.edu/tid/nvn40c00.
62. Covington MW. INFOTAB. 1982. Economic impact studies. 07 Jun. Philip Morris. http://legacy.library.ucsf.edu/tid/atn97e00.
63. Verkerk HG. 1985. INFOTAB regional workshop - Kuala Lumpur May 13-15, 1985 international overview of main issues. 13 May. Tobacco Institute. http://legacy.library.ucsf.edu/tid/jjq52f00.
64. INFOTAB. 1982. INFOTAB ten nation study. 13 Jul. RJ Reynolds. http://legacy.library.ucsf.edu/tid/uzz19d00.
65. Corti A. INFOTAB. 1982. Ten nation study. 23 Sep. Brown & Williamson. http://legacy.library.ucsf.edu/tid/eqk02d00.
66. Marcotullio RJ. 1982. INFOTAB advisory group meeting - July 14/15, 1982 Baden-Baden, Germany. 19 Jul. RJ Reynolds. http://legacy.library.ucsf.edu/tid/ozz19d00.
67. 1983. Presentation on social costs/social values to INFOTAB Board of Directors meeting October 31, 1983. 31 Oct. RJ Reynolds. http://legacy.library.ucsf.edu/tid/epk68d00.
68. Kloepfer W. 1984. [The cost of the monograph commissioned by INFOTAB]. 31 Oct. Tobacco Institute. Minnesota AG. http://legacy.library.ucsf.edu/tid/lga52f00.
69. Littlechild SC. 1984. [Letter from SC Littlechild to B Simpson]. 19 Nov. http://tobaccodocuments.org/pm/2023272536.html.
70. Vogel C. Reemtsma GmbH. 1981. Minutes of the first meeting of the project team 'Social Values' in Paris, October 16, 1981 16 Oct. http://tobaccodocuments.org/rjr/502091039-1041_D1.html.
71. Marcotullio RJ. 1982. INFOTAB advisory group meeting - Miami November 28 - 30 1982. 08 Dec. RJ Reynolds. http://legacy.library.ucsf.edu/tid/gzz19d00.

From: McDaniel PA, Intinarelli G, Malone RE: **Tobacco industry issues management organizations: Creating a global corporate network to undermine public health,** *Globalization and Health,* 2007.

72. [INFOTAB]. 1981. Project proposal identify and orient experts on public smoking in less developed countries. 28 Dec. Philip Morris. http://legacy.library.ucsf.edu/tid/hwk87e00.
73. Barnoya J, Glantz S: Tobacco industry success in preventing regulation of secondhand smoke in Latin America: the "Latin Project". *Tob Control* 2002, 11(4):305-314.
74. Marcotullio RJ. 1981. INFOTAB board of directors meeting - London, March 30, 1981. 06 Apr. RJ Reynolds. http://legacy.library.ucsf.edu/tid/zke29d00.
75. Corradini R. INFOTAB. 1984. Report from the secretariat. 22 May. RJ Reynolds. http://legacy.library.ucsf.edu/tid/txq85d00.
76. Wells III JK. 1982. INFOTAB Advisory Council Meeting July 14 and 15, 1982. 21 Jul. Brown & Williamson. http://legacy.library.ucsf.edu/tid/tez95a00.
77. [INFOTAB]. 1983. The five arguments against tobacco advertising censorship. Philip Morris. http://legacy.library.ucsf.edu/tid/cbs74e00.
78. Marcotullio RJ. 1984. INFOTAB advisory group meeting Brussels, February 20-22, 1984. 09 Mar. RJ Reynolds. http://legacy.library.ucsf.edu/tid/ouu75d00.
79. Marcotullio RJ. 1982. INFOTAB board of directors meeting, April 2, 1982 Brussels. 02 Apr. RJ Reynolds. http://legacy.library.ucsf.edu/tid/qke29d00.
80. King TCH. INFOTAB. 1989. CRU/IAA study "Juvenile Smoking Initiation and Advertising". 18 Apr. Tobacco Institute. Minnesota AG. http://legacy.library.ucsf.edu/tid/vqd52f00.
81. Covington MW. 1982. INFOTAB public smoking project. 07 Jul. Brown & Williamson. http://legacy.library.ucsf.edu/tid/ijc01c00.
82. Marcotullio RJ. 1984. INFOTAB advisory group meeting, Brussels, April 4, 1984. 09 Apr. RJ Reynolds. http://legacy.library.ucsf.edu/tid/gzq77c00.
83. Corti A. INFOTAB. 1983. INFOTAB meeting of the board of directors Bath 831030 - 831031. 22 Nov. Philip Morris. http://legacy.library.ucsf.edu/tid/vtz46e00.
84. INFOTAB. 1984. The CATAX pack. 18 Oct. RJ Reynolds. http://legacy.library.ucsf.edu/tid/jvv44d00.
85. Tobacco Institute. 1982. Communication Commitee meeting. 12 Nov. http://tobaccodocuments.org/lor/04234700-4701.html.
86. INFOTAB. 1984. INFOTAB catalogue of audio-visual material. Jul. Brown & Williamson. http://legacy.library.ucsf.edu/tid/rhl02d00.
87. Corti A. 1983. Advisory group meeting Brussels, Jan 13/14 1983 draft minutes. 13 Jan. Brown & Williamson. http://legacy.library.ucsf.edu/tid/ial02d00.
88. Verkerk HG. INFOTAB. 1983. Fifth World Conference on Smoking and Health Winnipeg - July 10-15, 1983. 09 Jun. Tobacco Institute. http://legacy.library.ucsf.edu/tid/cny62f00.
89. Verkerk HG. INFOTAB. 1983. Fifth World Conference on Smoking and Health Winnipeg, July 10-15, 1983. 20 Jun. Tobacco Institute. http://legacy.library.ucsf.edu/tid/kmy62f00.
90. INFOTAB. 1983. Final report; fifth World Conference on Smoking and Health Winnipeg, Canada, July 10-15, 1983. Jul. Tobacco Institute. Minnesota AG. http://legacy.library.ucsf.edu/tid/trx82f00.
91. Simpson B. INFOTAB. 1984. Passive Smoking Assessment Group. 29 Nov. British American Tobacco. http://bat.library.ucsf.edu//tid/yoj00a99.
92. [Corti A]. INFOTAB. 1984. Project proposal to advisory group public/passive smoking. 02 Feb. http://tobaccodocuments.org/batco/109879792-9798.html.
93. INFOTAB. Report and proposals-"passive" smoking assessment. 1984 (est.). British American Tobacco. http://bat.library.ucsf.edu//tid/zoj00a99.
94. Grice H. Tobacco Advisory Council. 1985. Passive smoking. 17 Jan. British American Tobacco. http://bat.library.ucsf.edu//tid/tib86a99.
95. Daniel J. Edelman Ltd. 1987. INFOTAB ETS project the overall plan. 12 Mar. RJ Reynolds. http://legacy.library.ucsf.edu/tid/aza87c00.

96. Simpson B. INFOTAB. 1987. Environmental tobacco smoke. 11 May. British American Tobacco. http://bat.library.ucsf.edu//tid/qvh00a99.
97. INFOTAB. 1987. Environmental tobacco smoke. Sep. http://tobaccodocuments.org/pm/2501251358-1566.html.
98. Verkerk H. INFOTAB. 1984. Restatement of Developing Countries Strategy Group activities. 22 Mar. Brown & Williamson. http://legacy.library.ucsf.edu/tid/lql02d00.
99. Developing Countries Strategy Group. 1983. The Rome presentation. 15 Nov. British American Tobacco. http://bat.library.ucsf.edu//tid/lbc44a99.
100. INFOTAB. 1984. A meeting of the board of directors of INFOTAB Essex House - New York. 20 Jul. Philip Morris. http://legacy.library.ucsf.edu/tid/znt24e00.
101. Oldman M. INFOTAB. 1989. Briefing meetings with ambassadors to the U.N. in Geneva and with permanent representatives to the F.A.O. in Rome. 20 Apr. Brown & Williamson. http://legacy.library.ucsf.edu/tid/dez31f00.
102. Simpson B. INFOTAB. 1984. Item 4 report from the secretary general. Philip Morris. http://legacy.library.ucsf.edu/tid/qoa81f00.
103. INFOTAB. 1984. European economic impact study. 12 Apr. British American Tobacco. http://bat.library.ucsf.edu//tid/huj47a99.
104. World Health Organization. 1982. Economic Aspects of Smoking. 22 Nov. Philip Morris. http://legacy.library.ucsf.edu/tid/xvv32e00
105. Marcotullio RJ. 1985. INFOTAB board of directors meeting, London, July 17/18, 1985. 22 Jul. RJ Reynolds. http://legacy.library.ucsf.edu/tid/ddb97c00.
106. Marcotullio RJ. 1984. INFOTAB advisory group meeting, Florida-June 5-7, 1984. 19 Jun. RJ Reynolds. http://legacy.library.ucsf.edu/tid/luu75d00.
107. Ogilvy & Mather. 1984. Courtesy campaign development for INFOTAB. RJ Reynolds. http://legacy.library.ucsf.edu/tid/mxv75d00.
108. Philip Morris. 1986. Minutes staff meeting 26 Aug. http://legacy.library.ucsf.edu/tid/rgv36e00.
109. INFOTAB. 1990. Seizing the initiative: action on environmental tobacco smoke. Oct. Philip Morris. http://legacy.library.ucsf.edu/tid/gzv19e00.
110. Wells III JK. 1989. [Minutes from the March meeting of the Global Issues Working Party]. 13 Apr. Brown & Williamson. http://legacy.library.ucsf.edu/tid/jpx70f00.
111. INFOTAB. 1987. Meeting of the steering committee, 6th World Conference on Smoking and Health. 24 Jun. British American Tobacco. http://bat.library.ucsf.edu//tid/aaf41a99.
112. Kloepfer B. 1987. Meeting reports TAC and INFOTAB. 27 May. Tobacco Institute. http://legacy.library.ucsf.edu/tid/yuj91f00.
113. Corti A. INFOTAB. 1987. Preliminary appraisal of 6th World conference on smoking and health, Tokyo. 12 Nov. Tobacco Institute. DOJ CIVIL. http://legacy.library.ucsf.edu/tid/kyl40c00.
114. INFOTAB. 1987. [Please find the attached daily summary of the 6th World Conference]. 11 Nov. Tobacco Institute. http://legacy.library.ucsf.edu/tid/pqs62f00.
115. Verkerk HG. INFOTAB. 1988. 6th World Conference on Smoking & Health - Tokyo, November 9-12, 1987. 29 Jan. Tobacco Institute. http://legacy.library.ucsf.edu/tid/mbu42f00.
116. Deroulez F. INFOTAB. 1989. International Public Affairs Committee meeting - Tuesday, 21st February, 1989 draft minutes. 21 Feb. Brown & Williamson. http://legacy.library.ucsf.edu/tid/hxl02d00.
117. Bloxcidge J. INFOTAB. 1991. ETS: Tobacco Institute of Australia (TIA) vs Australian Federation of Consumer Organization (AFCO) - advocacy advertising judgment. 07 Feb. British American Tobacco. http://bat.library.ucsf.edu//tid/gzt24a99.
118. INFOTAB. 1990. INFOTAB 1990 senior management briefing. Brown & Williamson. http://legacy.library.ucsf.edu/tid/apx70f00.

From: McDaniel PA, Intinarelli G, Malone RE: **Tobacco industry issues management organizations: Creating a global corporate network to undermine public health,** *Globalization and Health,* 2007.

119. INFOTAB. 1989 (est.). Proposed scheme of membership - international leaf dealers. British American Tobacco. http://bat.library.ucsf.edu//tid/zrf56a99.

120. INFOTAB. 1990. Background material for enquiries received during the Seventh World Conference on Tobacco and Health. Mar. Tobacco Institute. Minnesota AG. http://legacy.library.ucsf.edu/tid/nfq52f00.

121. King TCH. INFOTAB. 1990. World Conference on Tobacco and Health Perth, Western Australia - 1st to 5th April 1990. 12 Mar. Tobacco Institute. http://legacy.library.ucsf.edu/tid/mfq52f00.

122. INFOTAB. 1990. World Conference on Tobacco and Health monitoring report. 04 Apr. Tobacco Institute. http://legacy.library.ucsf.edu/tid/weq52f00.

123. INFOTAB. 1990. Global Argumentation Project; minutes of the meeting held on 30th January 1990 at the Tobacco Institute, Washington DC. 30 Jan. Tobacco Institute. Minnesota AG. http://legacy.library.ucsf.edu/tid/vjj52f00.

124. Loader R. INFOTAB. 1990. Global Argumentation Project. 18 Apr. Tobacco Institute. http://legacy.library.ucsf.edu/tid/sqi52f00.

125. Warren MS. Shook Hardy & Bacon. 1991. Enclosed please find the draft pesticide position paper that was prepared by our office as part of the Global Argumentation Project. 02 May. RJ Reynolds. http://legacy.library.ucsf.edu/tid/bum51c00.
